# EHMTI-0331. Q-No: a questionnaire to predict nocebo in outpatients seeking neurological consultation

**DOI:** 10.1186/1129-2377-15-S1-D14

**Published:** 2014-09-18

**Authors:** C Deligianni, DD Mitsikostas

**Affiliations:** 1 Neurological Department, Athens Naval Hospital, Athens, Greece

## Background and aim

Nocebo affects significantly adherence and treatment outcome and varies considerably among neurological conditions. We aimed to evaluate a questionnaire to predict nocebo in outpatients seeking neurological consultation.

## Methods

A four-item (rating range 0-20) self-fulfilled questionnaire (Q-No) was given in outpatients seeking neurological consultation at the Athens Naval Hospital. A blind to Q-No scoring neurologist rated outpatients as nocebo or no-nocebo after follow-up of >6 months.

## Results

338 (71.6% females) patients with mean age 46.9 (±13.8) years fulfilled the Q-No and the mean total score was 13.2 (±3.7). The Crombach's alpha coefficient was 0.627. Neurologist suggested 80 patients (23.7%) as nocebo and 258 as no-nocebo (mean Q-No score=12.4 95% CI: [12.0-12.9] and 15.8, 95%CI: [15.1-16.6], respectively). By using a cut-off at score 16 the Q-No predicts nocebo with 82.6% specificity and 61.3% sensitivity.

## Conclusions

Q-No may serve as a useful tool to predict nocebo in outpatients seeking neurological consultation.

No conflict of interest.

